# A new insight into pachychoroid diseases: Remodeling of choroidal vasculature

**DOI:** 10.1007/s00417-022-05687-6

**Published:** 2022-05-16

**Authors:** Shoji Kishi, Hidetaka Matsumoto

**Affiliations:** 1 Maebashi Central Eye Clinic, 2-54-5 Shimokoide, Maebashi, Gunma 371-0031 Japan; 2 Department of Ophthalmology, Gunma University Graduate School of Medicine, 3-39-15, showa, Maebashi, Gunma 371-8511 Japan

**Keywords:** Central serous chorioretinopathy, Pachychoroid disease, Pachyvessel, Venous anastomosis, Vortex vein, Watershed zone

## Abstract

**Purpose:**

Pachychoroid spectrum diseases are regarded as being different manifestations of a common pathogenic process. We suggest that pachychoroid diseases are consequences of chronic vortex vein stasis.

**Methods:**

We describe how we came to this conclusion based on our own recent reports as well as a search of the related literature.

**Results:**

Central serous chorioretinopathy (CSC) is the first stage of pachychoroid spectrum diseases. CSC is caused by congestion of choroidal veins, which are branches of the vortex veins. The venous outflow tract of the choroid is divided into four quadrants, based on horizontal and vertical watershed zones, with one or two vortex veins in each quadrant being independently responsible for venous outflow. In acute CSC, vortex vein stasis frequently causes asymmetric dilatation of the vortex veins in the horizontal watershed. The area of geographic filling delay in the choriocapillaris coincides with the area of this asymmetrically dilated vortex veins. With chronic stasis of the vortex veins, venous anastomosis occurs in the watershed zone as a means of compensating for the stasis, and the choriocapillaris becomes occluded in the area of filling delay. The anastomotic vessels dilate, becoming often hyperpermeable, and are then recognizable as pachyvessels. With the development of choriocapillaris ischemia, choroidal neovascularization (CNV) occurs at the site of pachyvessels. This is termed pachychoroid neovasculopathy (PNV). Polypoidal choroidal vasculopathy is regarded as a variant of PNV.

**Conclusions:**

Intervortex venous anastomosis is among the key factors underlying the development of pachychoroid diseases. Remodeling of the venous drainage route though the anastomosis across the watershed zones is apparently a common response to chronic vortex vein stasis. 
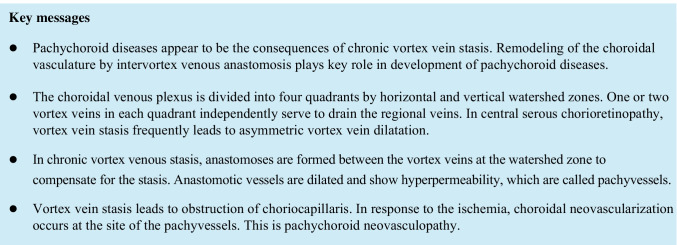

## The mystery of CSC

**Fig. 1:**
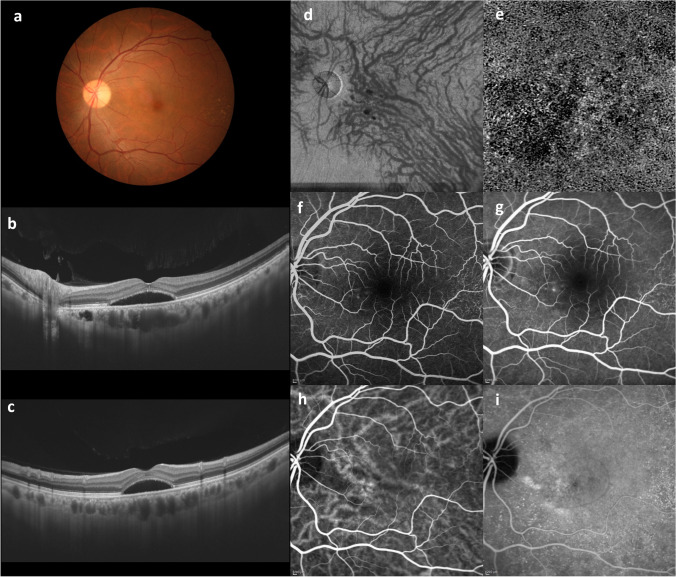
Characteristics of pachychoroid diseases. An illustrative case: 53-year-old man with central serous chorioretinopathy. **a** Color fundus photograph shows a serous retinal detachment (SRD) in the macula. **b** and **c** Horizontal and vertical 12-mm B-mode ocular coherence tomography (OCT) images through the fovea show pachychoroid with dilated outer choroidal vessels (vortex veins) associated with the SRD. The central choroidal thickness is 361 mm. **d** En face OCT image (12 × 12 mm) shows dilated vortex veins in the deep layer of the choroid. The horizontal watershed is lost due to the anastomoses between the superior and inferior vortex veins. **e** OCT angiography (3 × 3 mm) shows a decreased flow signal of choriocapillaris in the macula. **f** and **g** Fluorescein angiography (early and late phases) shows dye leakage within the SRD area. **h** and **i** Indocyanine green angiography (early and late phases) shows dilated choroidal vessels with hyperpermeability (pachyvessels) between the papilla and macula. Reproduced with permission from reference 31

Fluorescein angiography (FA) has demonstrated dye leakage from the retinal pigment epithelium (RPE) and accumulation at sites of serous retinal detachment characteristic of central serous chorioretinopathy (CSC). In the era when mainly FA was used for diagnosis, CSC was thought to be caused by disruption of the barrier function of the RPE. In the 1990s, indocyanine green angiography (ICGA) came into widespread use and revealed delayed filling of the choriocapillaris as well as both dilatation and hyperpermeability of the choroidal veins (Fig. 1). The leakage point from the RPE was directly above or near a hyperpermeable and dilated choroidal vein. Even when a serous retinal detachment heals in response to laser photocoagulation of the leakage point, the background choroidal hyperpermeability and vasodilation do not subside, such that CSC can recur at the area of choroidal hyperpermeability [1]. These observations supported the consensus view that CSC is attributable to choroidal venous congestion. Although age-related macular degeneration (AMD) and CSC were long thought to be entirely different and unrelated diseases, polypoid choroidal vasculopathy (PCV) has been shown to have similarities with CSC in terms of increased choroidal permeability and choroidal thickening. In 2000, Yannuzzi and colleagues reported that PCV can present as CSC and that it is important to detect polypoidal choroidal neovascularization (CNV) on ICGA to differentiate between these two clinical entities [2]. Furthermore, approximately 10–15% of patients with PCV also have a history of CSC, while this disorder is rare in typical AMD cases [3, 4].

## The pachychoroid disease concept

**Fig. 2:**
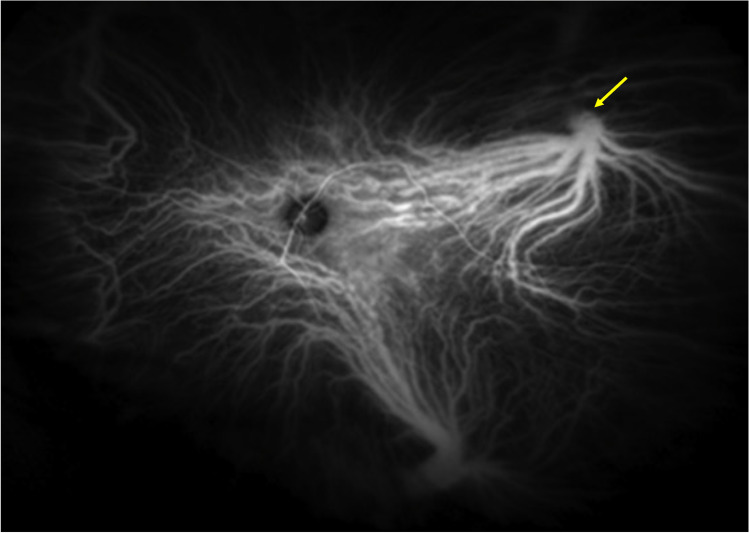
Ultra-widefield indocyanine green angiography on an eye with central serous chorioretinopathy shows dilatation of the superotemporal vortex vein from its distal end to the ampulla (arrow). Reproduced with permission from reference 13

ICGA revealed choroidal vascular dilatation and increased permeability to be present in both PCV [5] and CSC [6]. Since 2012, when it became possible to visualize the choroid on optical coherence tomography (OCT), researchers have recognized that there is a common disease spectrum of choroidal thickening, including dilated choroidal vessels in Haller’s layer, thinning of the choriocapillaris and Sattler’s layer, and abnormalities of the RPE over the pachyvessels [7–10]. OCT angiography revealed a decrease in the flow signal of the choriocapillaris [11]. Freund and colleagues defined these disorders as pachychoroid spectrum diseases, which encompass CSC, pachychoroid pigment epitheliopathy [7], pachychoroid neovasculopathy (PNV) [8], PCV [9], and peripapillary pachychoroid syndrome [10]. Cheung and colleagues reviewed 115 pachychoroid-related articles published through 2019 [12]. They described the characteristics of pachychoroid diseases (Fig. 2). However, the following issues have yet to be resolved: (1) what are pachyvessels? Choroidal thickening is due to dilatation of the choroidal vessels in Haller’s layer, which are referred to as “pachyvessels.” On en face OCT, the pachyvessels are detected in the outer layer of the choroid. They do not show tapering toward the posterior pole. Pachyvessels tend to be clustered in the macular area. Pachyvessels often show choroidal vascular hyperpermeability on ICGA. (2) What causes inner choroidal layer thinning? Simple thickening of the choroid is also observed in healthy eyes. The hallmarks of pachychoroid disease are dilated Haller vessels and thinning of both the Sattler’s layer and the choriocapillaris. In some cases, pachyvessels occupy almost the entire choroidal layer associated with thinning of inner choroidal layer. These characteristic choroidal findings of pachychoroid disease are believed to represent distinct phenotypes with a common etiology. However, the underlying contributory factors remain essentially unknown.

## Ultra-widefield ICGA reveals dilated vortex vein ampulla

**Fig. 3:**
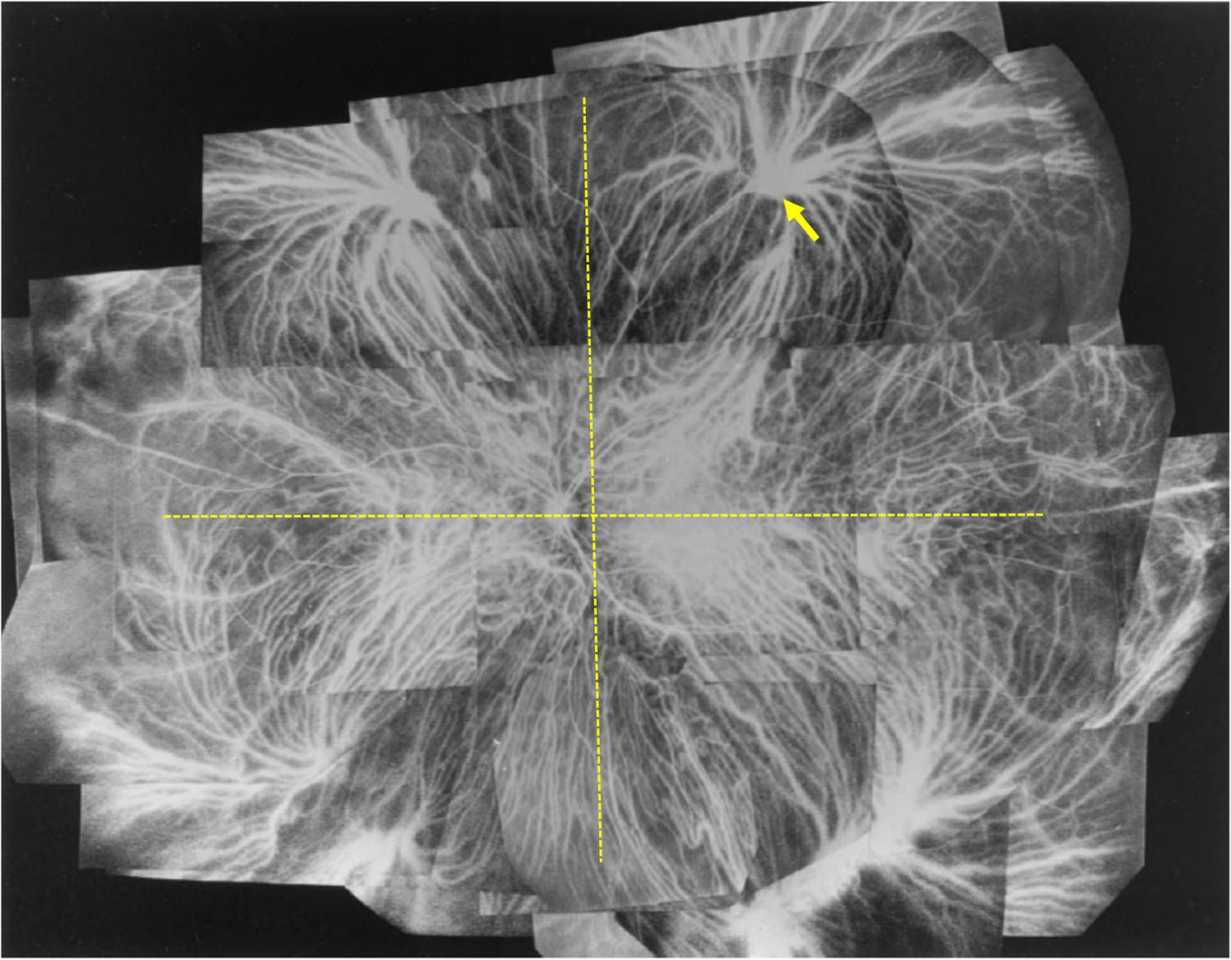
Wide-angle indocyanine green angiogram of the normal left eye of a 32-year-old volunteer is shown. Choroidal drainage routes are divided in four quadrants based on the horizontal and vertical watershed zones (dashed line). Venous blood in each quadrant drains into the corresponding regional vortex veins. Many choroidal veins merge into an ampulla of the vortex vein (arrow). Reproduced with permission from reference 25

Pang and colleagues reported ultra-widefield ICGA allowing the entire vortex vein in CSC to be visualized (Fig. 3) [13]. The affected vortex veins showed dilatation and hyperpermeability, converging to the dilated ampulla. These findings indicate that the dilated choroidal veins of the posterior fundus, as seen on conventional ICGA, are in fact branches of the vortex vein. The dilated ampulla suggests stasis of the vortex vein to be caused by an obstruction to its passage through the sclera. In the equatorial region via which the vortex vein passes through the sclera, the thickness of the sclera is 0.5 mm, but the vortex vein penetrates obliquely, such that the passage is actually 4 mm [14]. This anatomical feature may explain why the vortex vein tends to be blocked in the sclera. In the setting of high myopia with a thin sclera, CSC rarely develops. In nanophthalmos with a thick sclera, however, uveal effusion, a fulminant form of CSC, can develop. It was recently reported that the sclera is thicker in eyes with CSC than in normal eyes [15], and that asymmetric vortex veins are more frequently observed in eyes with a short axial length [16]. Stress has been reported to play a role in the development and exacerbation of CSC [17]. We speculate that vortex vein stasis is the underlying pathogenesis of CSC, and if the choroidal blood flow increases due to elevated sympathetic stimulation associated with stress, it can promote both the onset and the exacerbation of CSC.

## Dilatation of asymmetric vortex vein in CSC

**Fig. 4:**
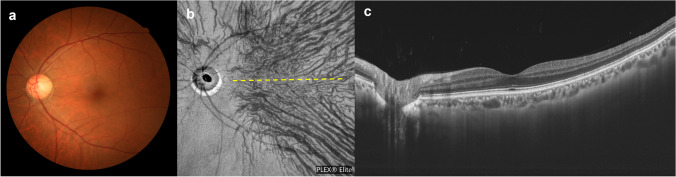
An illustrative case: images from a normal eye of a 56-year-old man. The refraction in the left eye was + 0.50 diopters. **a** The color fundus photograph shows a normal fundus appearance. **b** En face optical coherence tomography image (12 mm × 12 mm) shows vortex veins in the deep layer of the choroid. Superior and inferior vortex veins are symmetrical and there is a horizontal watershed zone (dashed line). **c** The 12-mm horizontal B-mode OCT image through the fovea shows that the retina and choroid both appear normal. The central choroidal thickness is 194 µm. Reproduced with permission from reference 29

**Fig. 5:**
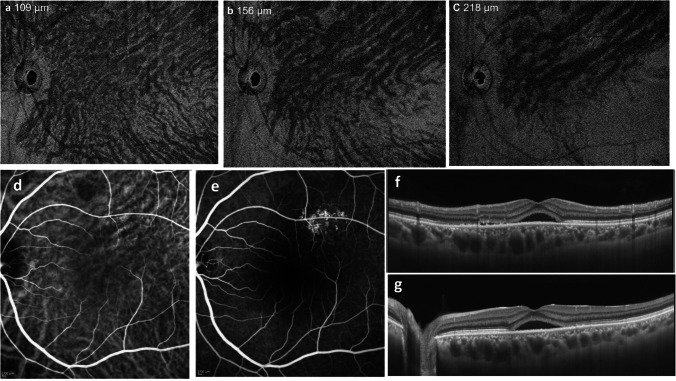
An asymmetric dilated vortex vein in central serous chorioretinopathy is shown. The left eye of a 48-year-old man with + 1.0 diopter of hyperopia. **a** En face OCT image in the superficial Haller’s layer at 109 μm from the Bruch membrane shows asymmetric superior vortex vein dilatation. **b** In the middle of the Haller’s layer at 156 μm from the Bruch membrane, asymmetric dilatation of the superior vortex vein is more clearly pronounced. Vortex vein branches do not taper toward the posterior pole. The horizontal watershed zone cannot be seen. **c** Deeper in the Haller’s layer, at 218 μm from the Bruch membrane. The superior vortex veins are dilated and retain their large calibers, terminating abruptly at their distal ends. **d** The venous phase of indocyanine green angiography (ICGA) shows hyperpermeability corresponding to the dilated superior vortex vein. **e** Fluorescein angiography shows hyperfluorescence of the retinal pigment epithelium in the area of hyperpermeability in ICGA images. **f** A vertical B-mode scan shows an enlarged vascular lumen of the choroid in the upper half of the posterior pole. **g** A horizontal B-mode scan shows dilated vortex veins at the horizontal watershed zone. Reproduced with permission from reference 19

Hayreh described that the venous outflow tract of the choroid is divided into four quadrants based on a horizontal and a vertical watershed zone, and noted that one or two vortex veins are independently responsible for the venous drainage in each of these quadrants [18]. A horizontal watershed zone passes through the macula and the papilla, a vertical one through the temporal side of the papilla (Fig. 4). Our research group used en face imaging with swept-source OCT to study the changes in superior and inferior vortex veins of the horizontal watershed zone characteristic of CSC. In normal eyes, both vortex veins are symmetrically distributed in 62% but are asymmetrical in 38%. They are not dilated and their distal ends show tapering at the watershed zone (Fig. 5). In CSC with unilateral vortex vein stasis, superior and inferior vortex veins are asymmetrical in terms of venous dilation and their distribution [19, 20]. These vortex veins do not taper toward the posterior pole, instead retaining their large calibers, and terminate abruptly. The watershed disappears, and the termini of the superior and inferior vortex veins often meet. Equally dilated bilateral vortex veins suggest bilateral vortex vein stasis. Vascular hyperpermeability occurs from dilated vortex veins close to the watershed zone, and asymmetric choroidal thickening seen on B-scan OCT coincides with the dilated vortex veins.

## Geographic filling delay of the choriocapillaris in CSC

**Fig. 6:**
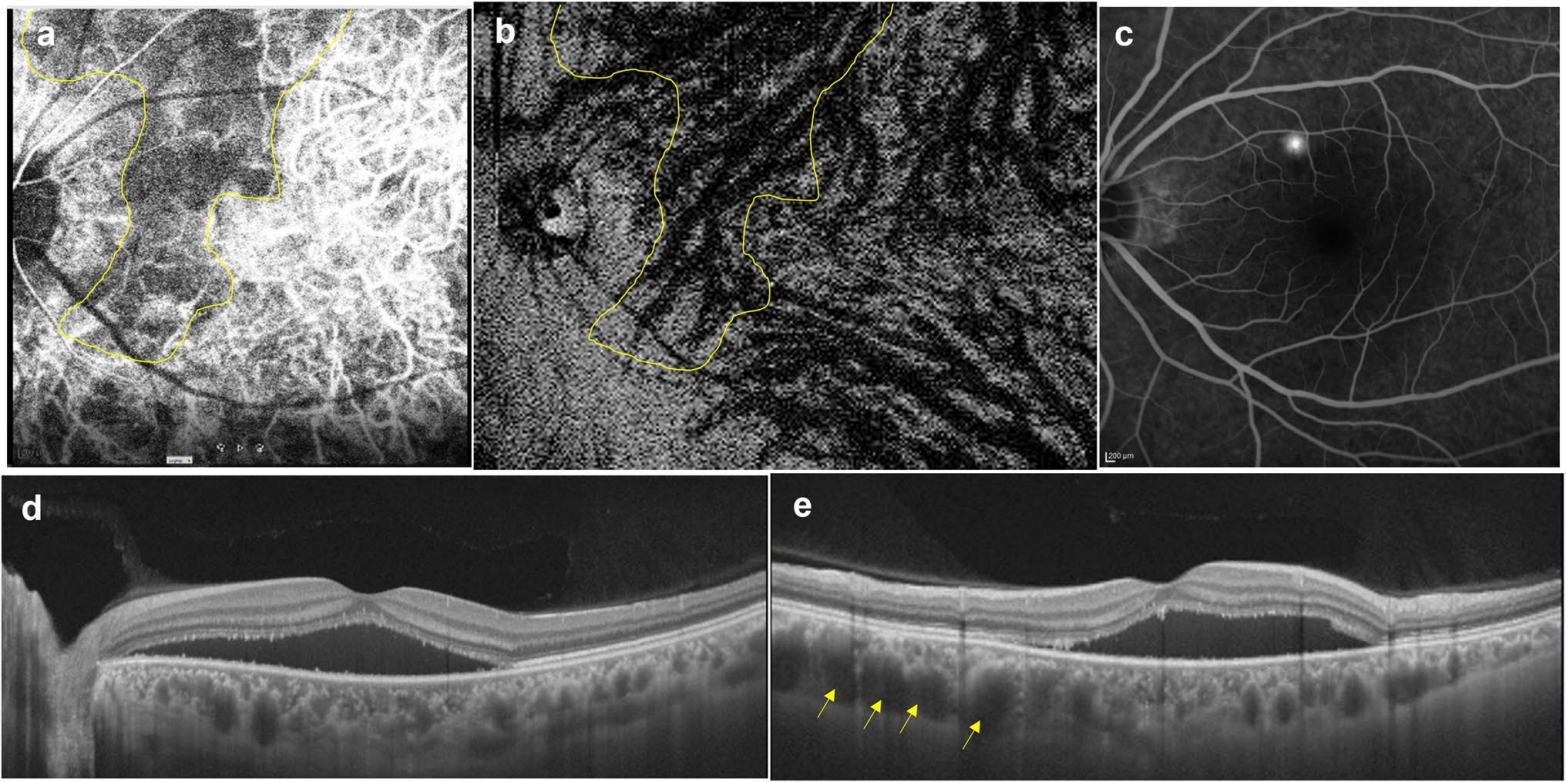
A 39-year-old man with acute central serous chorioretinopathy. **a** Early-phase indocyanine green angiography (ICGA) shows a geographic area (yellow line) corresponding to a filling delay in the choriocapillaris. **b** En face OCT image of the Haller’s layer of the choroid shows an asymmetrically dilated superior vortex vein the location of which (yellow line) corresponds to the geographic filling delay on ICGA. **c** Fluorescein angiography shows dye leakage from the region of a dilated vortex vein. **d** A horizontal B-mode scan shows serous retinal detachment with pachychoroid (central choroidal thickness; 459 μm). **e** A vertical B-mode scan shows marked dilatation of the superior vortex vein (arrows). Reproduced with permission from reference 21

**Fig. 7:**
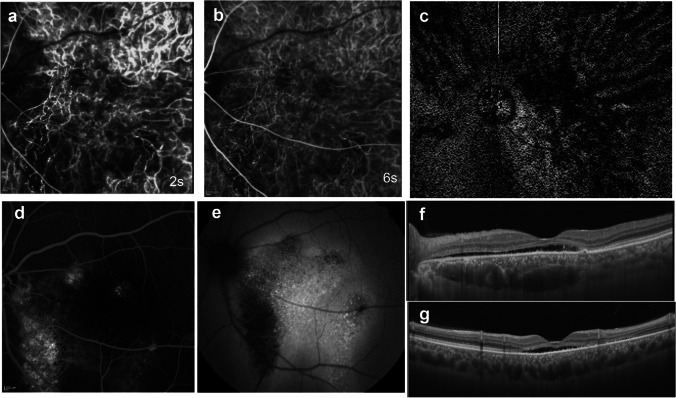
A 48-year-old man with chronic central serous chorioretinopathy. **a** Early-phase indocyanine green angiography (ICGA) shows a geographic area of filling delay in the choriocapillaris. **b** The choroidal filling delay persists in the venous phase. **c** En face OCT image shows anastomosis between superior and inferior vortex veins in the macular area. Anastomotic vessels and surrounding vortex veins show dilatation. The region of these dilated vessels corresponds to the area of the filling delay on ICGA. **d** Late-phase fluorescein angiography shows leakage above the macula and hyperfluorescence in the descending tract. **e** Fundus autofluorescence shows hyperfluorescent dots in the area of serous retinal detachment and hypofluorescence in the descending tract. **f** A horizontal OCT B-mode scan shows serous retinal detachment with dilated vortex and anastomotic veins between the papilla and the macula. **g** A vertical B-mode scan shows dilated vortex veins. Reproduced with permission from reference 21

Geographic filling delay of the choriocapillaris is known to occur in the early phase of ICGA in eyes with CSC. We reported that this filling delay area overlaps with the dilated vortex vein region in en face OCT images [21]. In eyes with acute CSC, the filling delay area was well-defined and corresponded to the dilated vortex vein region (Fig. 6). In eyes with chronic CSC, the choriocapillaris filling delay persists in the late phase of ICGA, suggesting occlusion of the choriocapillaris. The correspondence between the filling delay area on ICGA and the dilated vortex vein area in the en face OCT images decreased (Fig. 7) [21]. This is attributable to the filling delay area becoming poorly demarcated.

## Venous anastomosis at watershed zone in CSC

In eyes with CSC, venous anastomosis occurs between the superior and inferior vortex veins at the watershed zone (Figs. 2 and 5). The anastomotic vessels were dilated and dilatation of Haller vessels was observed on OCT B scans. Comparison of acute CSC and chronic CSC revealed the macular choroid to be significantly thinner in eyes with chronic CSC [21]. Patients with chronic CSC were 15 years older, on average, than those with acute CSC. The anastomosis at the watershed may have compensated for the vortex vein stasis and reduced choroidal thickness. Contact between the termini of superior and inferior vortex veins was frequent in normal eyes and also in those with acute CSC [19, 22]. The functions of these anastomotic vessels merit further research.

## Patterns of venous anastomosis

The horizontal watershed zone passes through both the papilla and the macula. Anastomoses between superior and inferior vortex veins generally occur in the macular area. The vertical watershed zone is just temporal to the papilla. Superotemporal and inferotemporal vortex veins may connect in the peripapillary area via anastomosis. These temporal vortex veins may on occasion connect with superior or inferior nasal vortex veins via another anastomosis in the vertical watershed zone. In such cases, the anastomotic vessels cluster in the peripapillary area. These dilated anastomotic vessels cause focal choroidal thickening between the papilla and the macula. This is thought to underlie peripapillary pachychoroid syndrome. PCV, with peripapillary dilated choroidal vessels from which choroidal neovascularization with polyp-like dilated tips arise, was reported [23]. Such vessels might be peripapillary anastomotic vessels.

## Pachyvessels appear to be anastomotic vessels

Comparison of the en face OCT images of anastomotic vessels with ICGA images revealed the anastomotic vessel to be dilated and have increased permeability. Newly formed anastomotic vessels appear to have a thin vascular wall and might thus be vulnerable to high venous pressure. Pachyvessels are considered to correspond to anastomotic vessels. In a PCV tissue specimen, there is reportedly a dilated choroidal vessel, thought to be a pachyvessel, the wall of which is thin and consists solely of endothelial cells [24].

## Intervortex venous anastomosis in eyes treated with scleral buckling

**Fig. 8:**
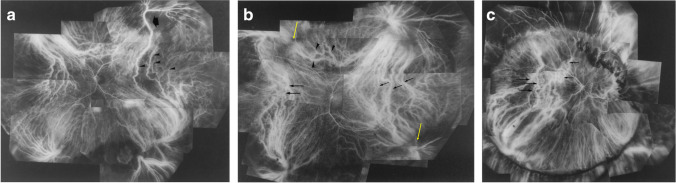
Composite indocyanine green angiogram of eyes that had undergone scleral buckling for retinal detachment. **a** An illustrative case: a 66-year-old man with retinal detachment 15 months after retinal detachment surgery. There is occlusion of the superotemporal vortex where the ampulla has disappeared (arrow). Blood from the anterior choroid and regional choroidal veins belonging to the occluded vortex vein flows via the new drainage routes (arrowheads) that connect to the inferotemporal vortex vein through venous anastomoses in the horizontal watershed zone. New drainage routes are dilated and show intense fluorescence. **b** An illustrative case: a 62-year-old man with retinal detachment 4 months after scleral encircling. The superonasal and inferotemporal vortex veins (yellow arrows) are both occluded at their ampulla. The venous blood in the inferotemporal quadrant drains into the superotemporal vortex vein through the venous anastomosis (black arrows) in the horizontal watershed zone. The superotemporal vortex vein is dilated. The venous blood in the superonasal quadrant drains partially to the inferonasal vortex vein through the anastomosis (black arrows) in the horizontal watershed and partially to the superotemporal vertex vein through the anastomosis (arrowheads) in the vertical watershed zone. **c** An illustrative case: the right eye of a 60-year-old woman with retinal detachment 9 years after scleral encircling. Vortex veins in the superior hemisphere of the fundus are occluded by scleral buckling with cryopexy. The inferotemporal vortex vein provides venous drainage from the superotemporal and superonasal quadrants. Long arrows indicate the anastomoses in the horizontal watershed zone from the superotemporal quadrant, and short arrows indicate the anastomoses in the vertical watershed zone from the superonasal area. Reproduced with permission from reference 25

Our research group reported remodeling of the choroidal vein drainage after a scleral buckling procedure for retinal detachment (Fig. 8) [25]. With occlusion of the superotemporal vortex vein, anastomosis developed in the horizontal watershed at the posterior pole, and venous blood then drained into the inferotemporal vortex vein. With occlusion of two or more vortex veins via scleral encircling, anastomoses formed in the vertical as well as at the horizontal watershed, and drainage of venous blood into the intact vortex veins was demonstrated. In both cases, the formation of dilated and tortuous anastomotic vessels in the macula was documented. The choroidal veins appeared to show marked plasticity, and with occlusion of the vortex vein there is remodeling of the outflow tract. Remodeling of the choroidal venous outflow tract has also been observed in eyes with radiation retinopathy and carotid cavernous fistula [26, 27].

## CNV arises at the site of pachyvessels

**Fig. 9:**
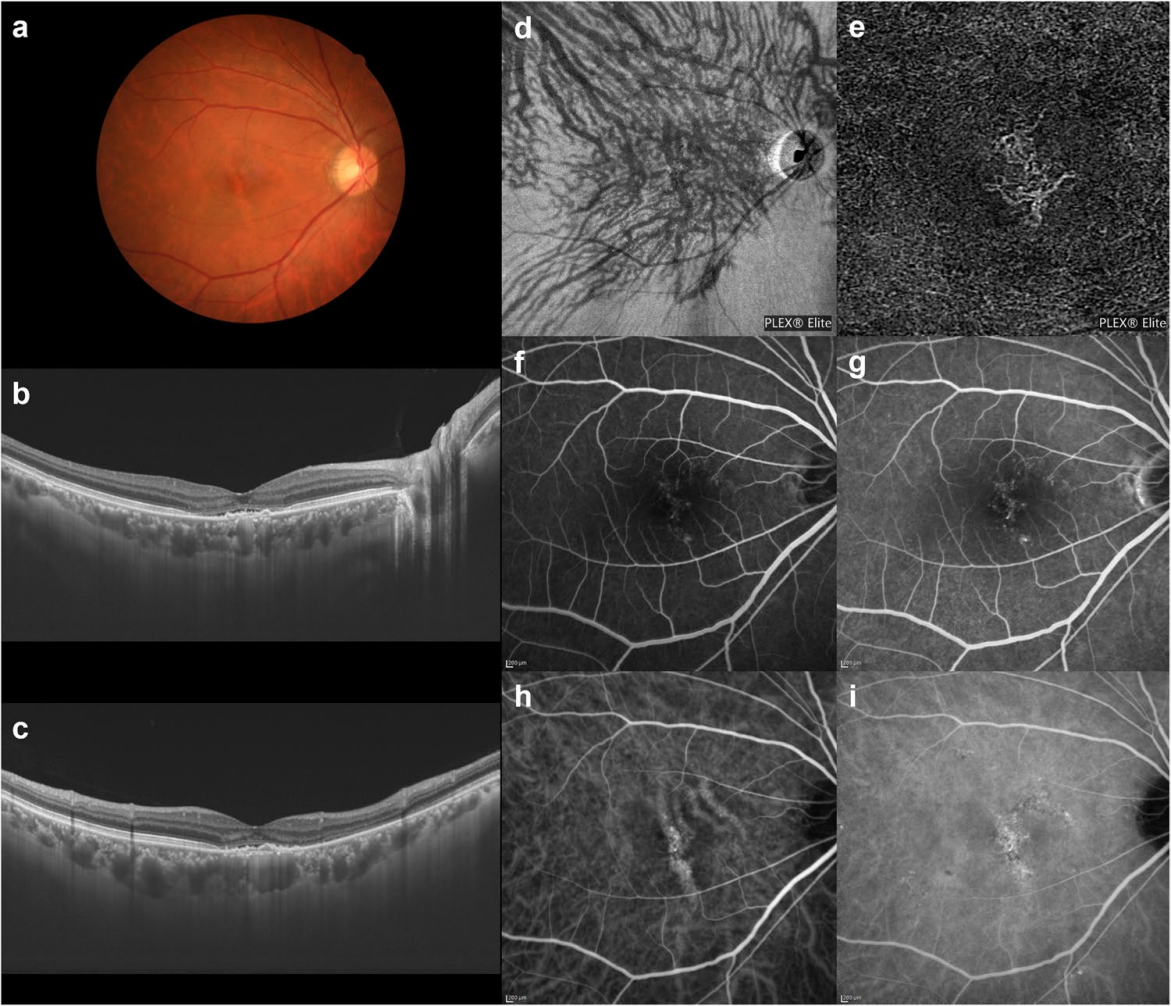
An illustrative case: images of an eye with pachychoroid neovasculopathy in a 53-year-old man. **a** Color fundus photograph shows a retinal pigment epithelium (RPE) abnormality at the fovea. There is a large choroidal vessel under the fovea. **b** and **c** The 12-mm horizontal and vertical B-mode optical coherence tomography (OCT) images through the fovea show pachychoroid with dilated outer choroidal vessels (vortex veins). A shallow irregular RPE detachment accompanied by slight serous retinal detachment is present at the fovea. The central choroidal thickness is 353 µm. **d** En face OCT image (12 mm × 12 mm) shows dilated vortex veins in the deep layer of the choroid. Superior vortex veins are markedly more evident than the inferior vortex veins. The horizontal watershed zone has disappeared, showing instead collateral veins due to anastomoses between the superior and inferior vortex veins. **e** OCT angiography (3 mm × 3 mm) shows network vessels corresponding to choroidal neovascularization (CNV) between the detached RPE and Bruch’s membrane. We detected CNV over the dilated vortex veins. **f** and **g** Fluorescein angiography (early and late phases) shows window defects and some oozing in the foveal area. **h** and **i** Indocyanine green angiography (early and late phases) shows dilated choroidal vessels with hyperpermeability in the macular area, raising suspicion of CNV in the fovea. Reproduced with permission from reference 29

**Fig. 10:**
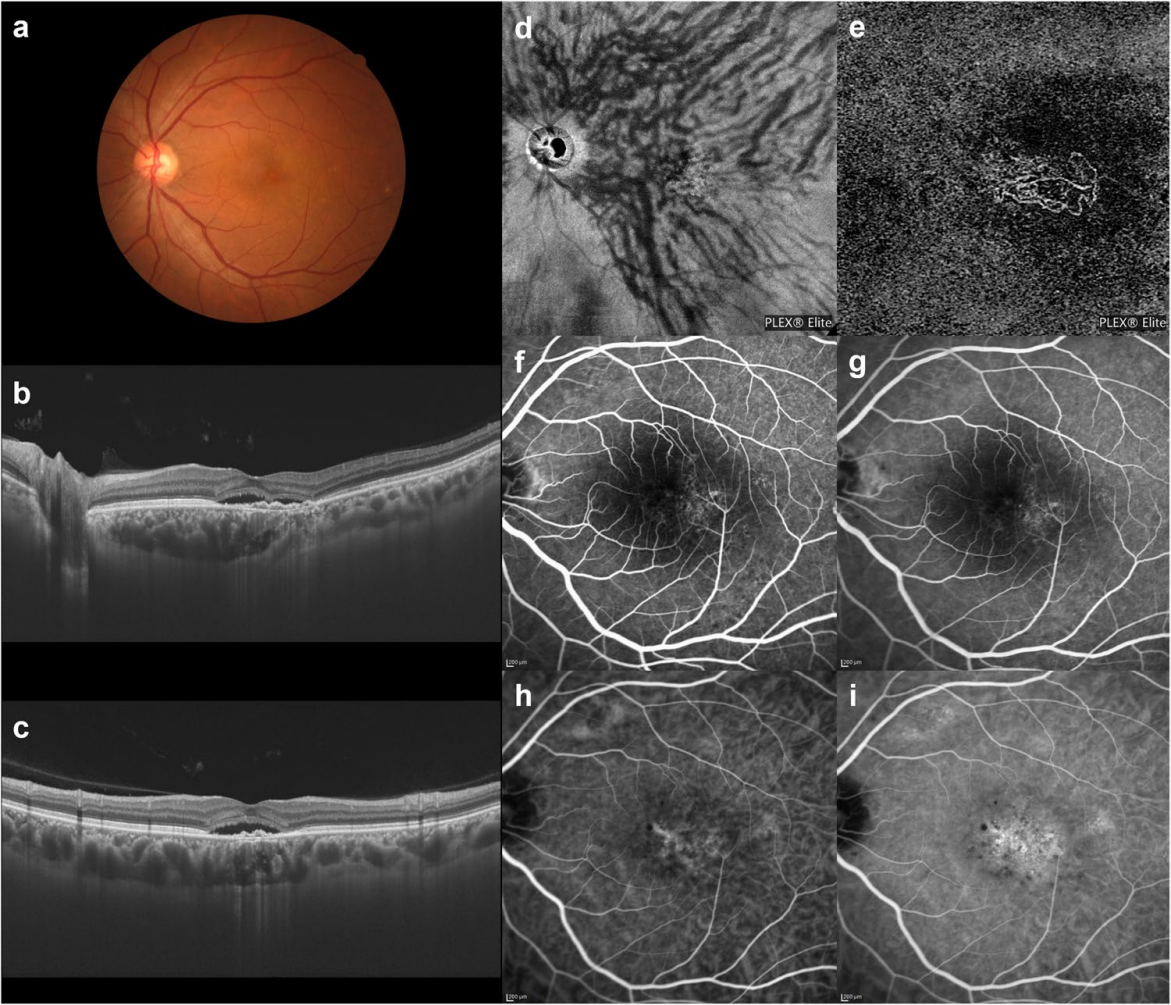
An illustrative case: images of an eye with pachychoroid neovasculopathy in a 54-year-old man. **a** The color fundus photograph shows a retinal pigment epithelium (RPE) abnormality in the macular area. **b** and **c** The 12-mm horizontal and vertical B-mode OCT images through the fovea show pachychoroid with dilated outer choroidal vessels (vortex veins). Dilated vortex veins are present between the papilla and subfovea in the horizontal B-mode OCT image. There is a shallow irregular RPE detachment accompanied by serous retinal detachment at the fovea. The central choroidal thickness is 386 µm. **d** En face OCT image (12 mm × 12 mm) showing dilated vortex veins in the deep layer of the choroid. Superior and inferior vortex veins are symmetrical. Venous anastomosis shows clustering in the peripapillary area. **e** OCT angiography (3 mm × 3 mm) demonstrates network vessels of choroidal neovascularization (CNV) between the detached RPE and Bruch’s membrane. CNV can be seen over the dilated anastomotic vessels. **f** and **g** Fluorescein angiography (early and late phases) shows window defects and some oozing in the macular area. **h** and **i** Indocyanine green angiography (early and late phases) shows findings raising suspicion of CNV at the fovea, as well as choroidal vascular hyperpermeability around the fovea. Reproduced with permission from reference 29

Chronic CSC is known to often be associated with retinal pigment epithelial detachment (PED). ICGA shows fluorescein dye leakage from PED. Since OCTA was introduced, CNV has often been detected in PED [28]. Chronic CSC with PED is thus frequently diagnosed as PNV. In eyes with PNV, as in those with CSC, venous anastomosis forms in the macular area and the peripapillary area in more than 90% of cases [29]. The anastomotic vessels show dilatation and hyperpermeability, findings collectively termed pachyvessels, as noted above. CNV arises from the pachyvessel site (Figs. 9 and 10). With OCT B-scanning, dilated pachyvessels can be seen in the choroid just beneath the macula. The Sattler’s layer of the inner choroid and the choriocapillaris are, however, very thin. This has been attributed to compression by the dilated Haller vessel, but we believe that choriocapillaris occlusion secondary to persistent vortex vein stasis leads to the thinning of the inner choroid. This ischemia may lead to CNV. We evaluated choroidal congestion using multimodal imaging in eyes with PNV [30]. All of the eyes studied showed choriocapillaris filling delay in the early phase of ICGA. The choriocapillaris filling delay areas broadly overlapped with that of dilated outer choroidal vessels. All eyes showed CNV localized within sites where choriocapillaris filling was delayed. RPE atrophy was present in 71 eyes (71.0%), of which 68 (95.8%) had RPE atrophy within the areas of choriocapillaris filling delay. These findings suggest chronic choriocapillaris ischemia secondary to vortex vein congestion to possibly lead to CNV development as well as RPE atrophy in eyes with PNV.

## Evolution of vascular changes in pachychoroid disease

We assessed patient age, central choroidal thickness (CCT), and vortex vein anastomosis in patients with pachychoroid diseases including CSC, PNV, and PCV (pachychoroid neovasculopathy with polypoidal lesions), for comparison with the values in normal controls matched for age and gender [31]. Patients with CSC were the youngest, followed by those with PNV, and then PCV. CSC eyes had the highest CCT values, followed by the PNV and then the PCV eyes. CCT was significantly greater in eyes with pachychoroid spectrum diseases than in healthy controls. Anastomosis between superior and inferior vortex veins was present in more than 90% of eyes with pachychoroid spectrum diseases, such that this finding was significantly more common than in healthy controls. Moreover, the mean diameter of vortex veins was significantly larger in eyes with CSC than in those with PCV [32]. These findings suggest congestion of vortex veins to possibly undergo gradual amelioration corresponding to anastomosis developing between the superior and inferior vortex veins as pachychoroid spectrum diseases progress.

**Fig. 11:**
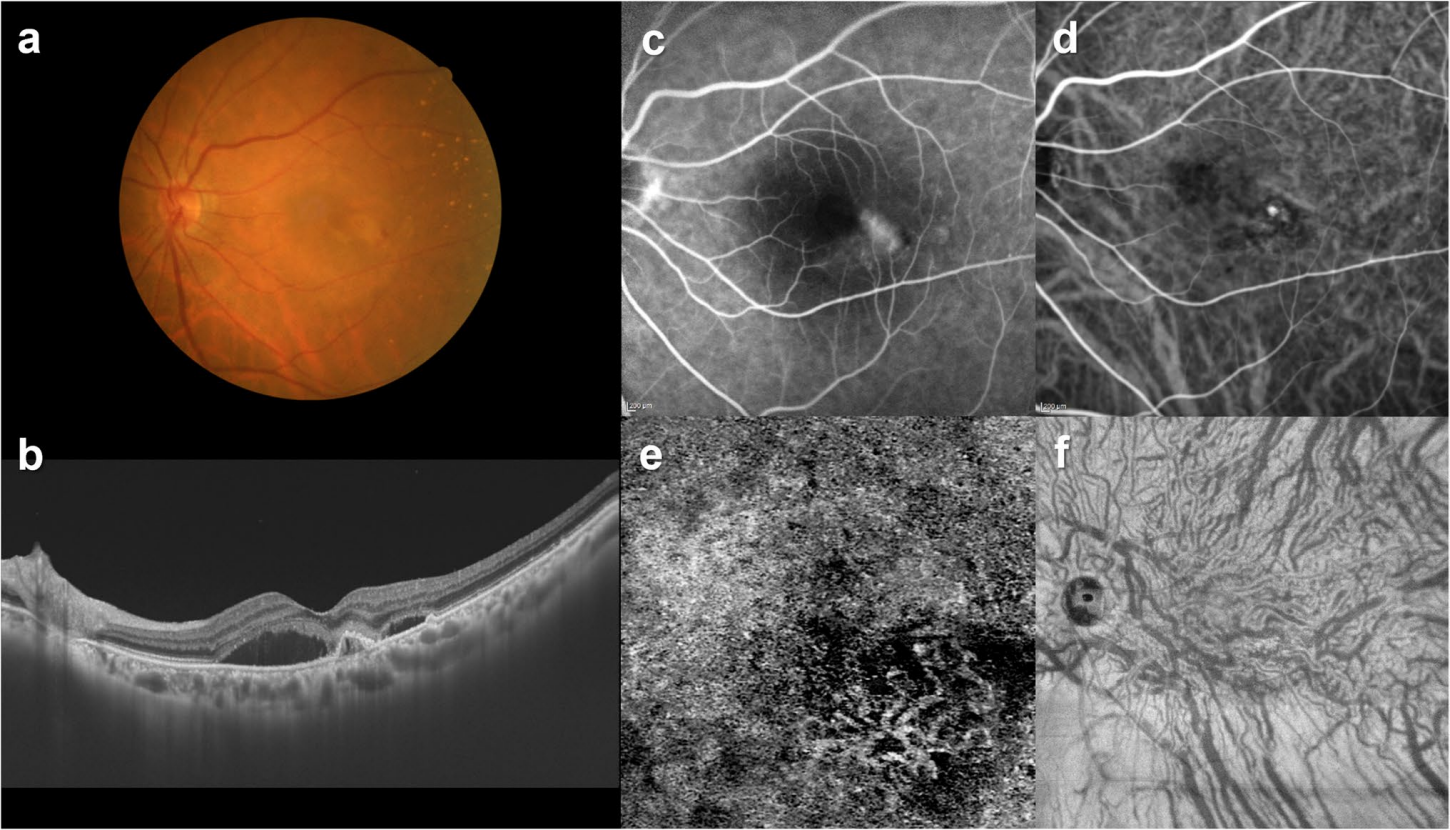
An illustrative case: images of the right eye of an 84-year-old female with polypoidal choroidal vasculopathy. **a** The color fundus photograph shows retinal pigment epithelium (RPE) detachments associated with subretinal hemorrhage and serous retinal detachment (SRD) in the macular area. **b** The 12-mm horizontal B-mode optical coherence tomography (OCT) images through the fovea show dilated outer choroidal vessels (vortex veins) accompanied by RPE detachment and SRD. The central choroidal thickness is 281 µm. **c** Fluorescein angiography shows window defects and leakage in the macular area. **d** Indocyanine green angiography shows dilated choroidal vessels and a polypoidal lesion temporal to the fovea. **e** OCT angiography (3 mm × 3 mm) shows network vessels accompanied by a polypoidal lesion between the detached RPE and Bruch’s membrane. **f** En face OCT image shows dilated vortex veins in the deep layer of the choroid. The horizontal watershed has been lost due to anastomoses between the superior and inferior vortex veins. Reproduced with permission from reference 33

**Fig. 12:**
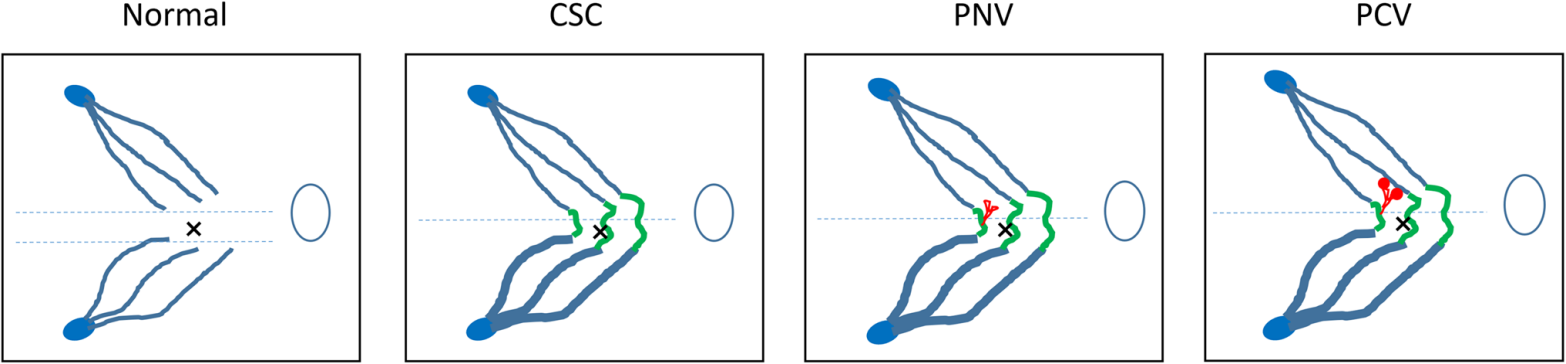
Diagrams of choroidal vasculature in a normal eye and eyes with pachychoroid spectrum diseases. Normal: superior and inferior vortex veins are symmetric at horizontal watershed zone. Vortex veins taper toward the posterior pole. CSC: asymmetric dilatation of superior and inferior vortex veins with loss of watershed. Venous anastomosis is seen at watershed in most cases (green). PNV: choroidal neovascularization arises at the site of anastomosis (red). Overlying choriocapillaris shows filling delay. PCV: The tip of choroidal neovascularization shows aneurysmal dilatation. CSC = central serous chorioretinopathy, PNV = pachychoroid neovasculopathy, PCV = polypoidal choroidal vasculopathy

On early-phase ICGA video, we detected pulsatile vortex venous flow in 76 eyes (25.8%) at the vortex veins connected with anastomosis between superior and inferior vortex veins in pachychoroid diseases (Fig. 11) [33]. PCV was recently recognized as a variant of PNV, which is considered to be a polyp-like extension of the tip of CNV of PNV. The choroid becomes even thinner, while the pachyvessel persists in the macular area. Because unlike the retina the choroid does not have a tissue-capillary complex, the CNV might be directly exposed to pulsatile blood flow. This may explain why the tip of the CNV becomes dilated into a polyp-like shape, which can lead to massive subretinal or sub-RPE hemorrhage (Fig. 12).

## Conclusions

Intervortex venous anastomosis is among the key factors underlying the development of pachychoroid diseases. Remodeling of the venous drainage route though the anastomosis across the watershed zones is apparently a common response to chronic vortex vein stasis. Vortex vein stasis leads to choriocapillaris filling delay, which in turns produces occlusion of the choriocapillaris. These changes are responsible for the characteristic features of pachychoroid diseases including thinning of the choriocapillaris and Sattler’s layer while pachyvessels (anastomotic vessels) become increasingly dilated. Ischemia secondary to occlusion of the choriocapillaris triggers the development of CNV. Our theory of anastomosis is becoming a new trend in the understanding of pachychoroid diseases [34–37].
